# The 7-aza­norbornane nucleus of epibatidine: 7-aza­bicyclo­[2.2.1]heptan-7-ium chloride

**DOI:** 10.1107/S2056989017012105

**Published:** 2017-08-30

**Authors:** Sergey N. Britvin, Andrey M. Rumyantsev

**Affiliations:** a Department of Crystallography, Saint-Petersburg State University, Universitetskaya Nab. 7/9, 199034 St Petersburg, Russian Federation; b Department of Genetics and Biotechnology, Saint-Petersburg State University, Universitetskaya Nab. 7/9, 199034 St Petersburg, Russian Federation

**Keywords:** crystal structure, cage compounds, nitro­gen heterocycles, amine, alkaloid, epibatidine

## Abstract

7-Aza­bicyclo­[2.2.1]heptane (7-aza­norbornane) is a bridged heterocyclic nucleus found in epibatidine, the alkaloid isolated from the skin of the tropical poison frog *Epipedobates tricolor*. Since epibatidine is known as one of the most potent acetyl­choline nicotinic receptor agonists, a plethora of literature has been devoted to this alkaloid. However, there are no structural data on the unsubstituted 7-aza­norbornane, the parent bicyclic ring of epibatidine and its derivatives. We herein present the structural characterization of the 7-aza­bicyclo­[2.2.1]heptane parent ring as its hydro­chloride salt, namely 7-aza­bicyclo­[2.2.1]heptan-7-ium chloride, C_6_H_12_N^+^·Cl^−^. The compete cation is generated by a crystallographic mirror plane with the N atom lying on the mirror, as does the chloride anion. In the crystal, the cations are linked to the anions by N—H⋯Cl hydrogen bonds, which generate [001] chains.

## Abstract 2

7-Aza­bicyclo­[2.2.1]heptane (7-aza­norbornane) is a bridged heterocyclic nucleus found in epibatidine. The structural characterization of the 7-aza­bicyclo­[2.2.1]heptane parent ring as its hydro­chloride salt, namely 7-aza­bicyclo­[2.2.1]heptan-7-ium chloride, has been carried out.

## Chemical context   

Since the discovery of the quinuclidine and tropane nuclei (Hamama *et al.*, 2006 ▸; Pollini *et al.*, 2006 ▸), elegant frameworks of bridged aza-heterocycles have been the focus of chemists exploring biologically active substances. One famous example in this series is epibatidine, (−)-2-(6-chloro­pyridin-3-yl)-7-aza­bicyclo­[2.2.1]heptane, an active component of the skin poison extracted from the small tropical frog *Epipedobates tricolor* (Spande *et al.*, 1992 ▸; Gerzanich *et al.*, 1995 ▸; Sullivan & Bannon, 1996 ▸; Dukat & Glennon, 2003 ▸). Epibatidine comprises the first natural example of a compound incorporating an 7-aza­bicyclo­[2.2.1]heptane (7-aza­norbornane) ring system (Fletcher *et al.*, 1994 ▸). Due to the extreme binding affinity of the *exo* isomer of epibatidine towards nicotinic acetyl­choline receptors, thousands of articles have been devoted to different aspects of its chemistry and biochemistry (see Carroll, 2004 ▸; Daly *et al.*, 2005 ▸; Yogeeswari *et al.*, 2006 ▸; Garraffo *et al.*, 2009 ▸). We are not aware, however, that an X-ray structure determination of the alkaloid itself has ever been reported, in spite of numerous publications related to its 
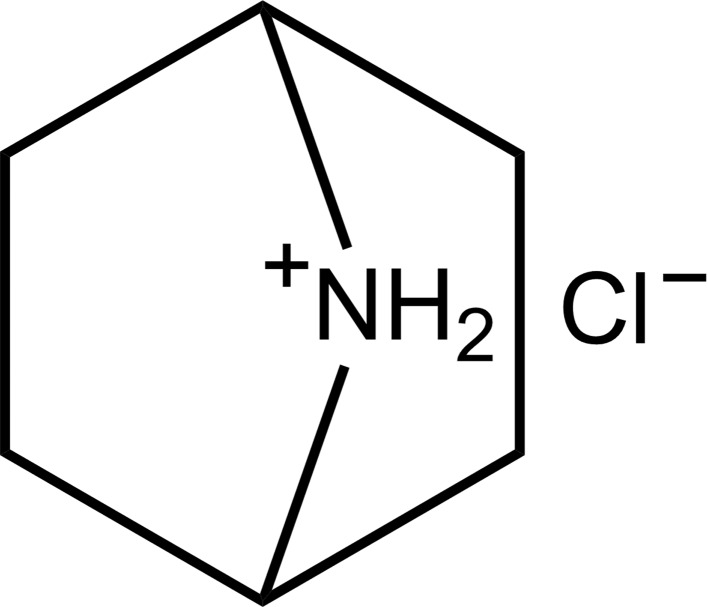
synthesis. Moreover, the mol­ecular structure of 7-aza­norbornane, the functional core of epibatidine, has also not been explored, in spite of the fact that 7-aza­norbornane has been known since 1930 (Braun & Schwarz, 1930 ▸; Fraser & Swingle, 1970 ▸). In continuation of our studies related to bridged aza-heterocyclic systems (Britvin *et al.*, 2015 ▸, 2016 ▸, 2017 ▸), we herein report on the structure of the unsubstituted 7-aza­bicyclo­[2.2.1]heptane parent ring as its hydro­chloride salt, namely 7-aza­bicyclo­[2.2.1]heptan-7-ium chloride, **1**.

## Structural commentary   

**Figure 1:**
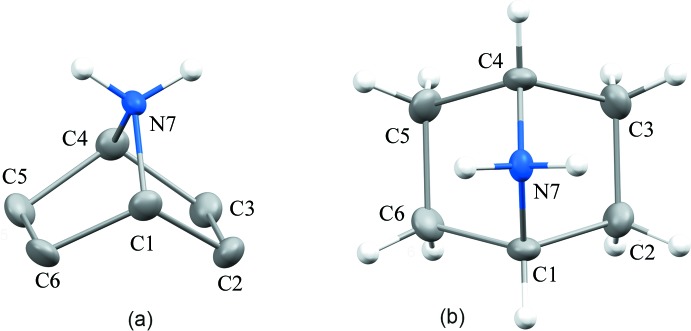
The molecular structure and systematic atomic numbering scheme of the 7-azabicyclo[2.2.1]heptane (7-azanorbornane) parent ring in **1**. Displacement ellipsoids are drawn at the 50% probability level. H atoms on C atoms in view (*a*) and the chloride counter-ion have been omitted for clarity. The labelling in the Figures corresponds to IUPAC notation (see text). Atoms C4, C3 and C5 are generated from C1, C2 and C6, respectively, by the symmetry operation (1 − *x*, *y*, *z*).

The parent ring of 7-aza­bicyclo­[2.2.1]heptane in **1** adopts a boat conformation (Fig. 1 ▸) resembling the molecular geometry of its nearest carbocyclic counterpart, bicyclo[2.2.1]heptane (norbornane), **2** (Fitch & Jobic, 1993 ▸). In order to achieve consistency of atomic labelling between the bicyclic cages of **1** and **2**, we herein apply the numbering scheme according to IUPAC nomenclature (Fig. 1) (Doms *et al.*, 1985 ▸). There are three unique C atoms (C1, C2 and C6) in the cation of **1**, with their clones C1^i^ [= C4 by IUPAC; symmetry code: (i) 1 − *x*, *y*, *z*], C2^i^ (= C3 by IUPAC) and C6^i^ (= C5 by IUPAC) generated by the mirror at *x* = 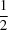. Inter­atomic distances between the respective framework sites of **1** are shorter compared with the corresponding values of **2**. The distances (Å) in **1** and **2** are: C1—C2 = 1.528 (2) and 1.551 (3), C1—C6 = 1.523 (3) and 1.578 (1), and C1—N7(C7) = 1.508 (2) and 1.551 (3). The C2^i^—C2—C1—C6 torsion angle determining the boat-like conformation is 109.4 (1)° in **1** and 108.7 (2)° in **2**. The s.u. values for **2** were generated using *PLATON* (Spek, 2009 ▸). Further details of the inter­atomic distances and angles of **1** can be found in the supporting information.

## Supra­molecular features   

**Figure 2:**
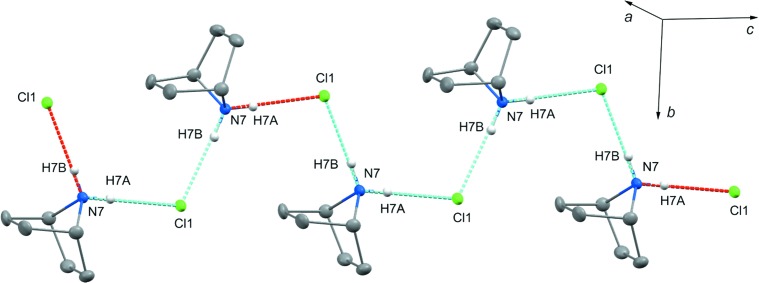
Hydrogen bonding in the crystal structure of **1**. Protonated mol­ecules of 7-aza­norbornane are linked *via* N—H⋯Cl hydrogen bonds to form infinite zigzag chains propagated along the *c* axis. Displacement ellipsoids are drawn at the 50% probability level. H atoms not involved in hydrogen bonding have been omitted for clarity.

The structural integrity of **1** is maintained *via* inter­molecular hydrogen bonding between the protonated secondary site N7 and the chloride counter-ion Cl1 (Table 1 ▸). Each chloride ion is linked to the two adjacent amine centres *via* N—H⋯Cl hydrogen bonds so that the 7-aza­norbornane cages are arranged into zigzag chains flattened on (010) and propagating along the *c*-axis direction (Fig. 2 ▸). That type of inter­leaved zigzag packing is known among chloride salts of secondary amines, both for alkyl- and aryl­amines (Adams *et al.*, 1997 ▸; Nancy *et al.*, 2003 ▸; Muller *et al.*, 2007 ▸) and heterocyclic systems (Gribkov *et al.*, 2006 ▸; Wang *et al.*, 2011 ▸; Fun *et al.*, 2011 ▸).

## Database survey   

Of more than 120 structures containing the 7-aza­norbornane ring system in the Cambridge Structural Database (CSD, Version 5.38, latest update May 2017; Groom *et al.*, 2016 ▸), 17 entries represent the 7-aza­bicyclo­[2.2.1]heptane parent ring unsubstituted at the carbon sites. All these compounds belong to *N*-substituted derivatives of 7-aza­norbornane (Ohwada *et al.* 1998 ▸; Cheng *et al.* 2002 ▸; Otani *et al.* 2003 ▸; Hori *et al.* 2008 ▸; Longobardi *et al.* 2015 ▸).

## Synthesis and crystallization   

7-Aza­bicyclo­[2.2.1]heptane hydro­chloride, **1**, was obtained from Sigma Aldrich. The purity of the substance has been proven by elemental analysis (analysis calculated for C_6_H_12_ClN: C 53.93, H 9.05, N 10.48%; found: C 53.89, H 9.08, N 10.44%). ^1^H NMR (400 MHz) spectrum (Bruker Avance 400, SiMe_4_ external standard, D_2_O solution): δ 4.21–4.19 (*m*, 2H, 2 × C*H* at C1 and C4; the atom-numbering scheme is according to IUPAC nomenclature, see Fig. 1 ▸), 1.92–1.84 (*m*, 4H, 4 × *endo*-*H*CH at C2, C3, C5, C6), 1.78–1.71 (*m*, 4H, 4 × *exo*-*H*CH at C2, C3, C5, C6). ^13^C{^1^H} NMR (101 MHz): δ 58.9 (*s*, C1 and C4), 26.7 (*s*, C2, C3, C5, C6). Crystals of **1** suitable for structural studies were obtained by slow evaporation of its aqueous solution.

## Refinement   

H atoms at the protonated N7 atom were refined freely, whereas H atoms on C atoms were refined based on a riding model. Crystal data, data collection and structure refinement details are summarized in Table 2 ▸.

## Supplementary Material

Crystal structure: contains datablock(s) I. DOI: 10.1107/S2056989017012105/zl2713sup1.cif

Structure factors: contains datablock(s) I. DOI: 10.1107/S2056989017012105/zl2713Isup2.hkl

Supporting information file. DOI: 10.1107/S2056989017012105/zl2713Isup3.mol

Supporting information file. DOI: 10.1107/S2056989017012105/zl2713Isup4.cml

CCDC reference: 1511233

Additional supporting information:  crystallographic information; 3D view; checkCIF report

## Figures and Tables

**Table 1:** Hydrogen-bond geometry (Å, °)

*D*—H⋯*A*	*D*—H	H⋯*A*	*D*⋯*A*	*D*—H⋯*A*
N7—H7*B*⋯Cl1^i^	0.88 (3)	2.25 (3)	3.127 (2)	175 (2)
N7—H7*A*⋯Cl1	0.87 (4)	2.25 (4)	3.122 (2)	178 (3)

Symmetry code: (i) 
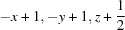
.

**Table 2:** Experimental details

Crystal data	
Chemical formula	C_6_H_12_N^+^·Cl^−^
*M*_r_	133.62
Crystal system, space group	Orthorhombic, *C* *m* *c*2_1_
Temperature (K)	100
*a*, *b*, *c* (Å)	9.1532 (6), 8.7029 (8), 8.7336 (5)
*V* (Å^3^)	695.71 (9)
*Z*	4
Radiation type	Mo *K*α
μ (mm^−1^)	0.45
Crystal size (mm)	0.08 × 0.06 × 0.04

Data collection	
Diffractometer	Bruker APEXII CCD
Absorption correction	Multi-scan (*SADABS*; Sheldrick, 2015 ▸)
No. of measured, independent and observed [*I* > 2σ(*I*)] reflections	3239, 777, 769
*R*_int_	0.017
(sin θ/λ)_max_ (Å^−1^)	0.638

Refinement	
*R*[*F* ^2^ > 2σ(*F* ^2^)], *wR*(*F* ^2^), *S*	0.017, 0.048, 1.15
No. of reflections	777
No. of parameters	47
No. of restraints	1
H-atom treatment	H atoms treated by a mixture of independent and constrained refinement
Δρ_max_, Δρ_min_ (e Å^−3^)	0.21, −0.12
Absolute structure	Refined as an inversion twin
Absolute structure parameter	0.19 (9)

Computer programs: *APEX2* (Bruker, 2015 ▸), *SAINT* (Bruker, 2015 ▸), *SHELXT* (Sheldrick, 2015 ▸a), *OLEX2* (Dolomanov *et al.*, 2009 ▸), *SHELXL2014* (Sheldrick, 2015 ▸b), *Mercury* (Macrae *et al.*, 2008 ▸) and *publCIF* (Westrip, 2010 ▸).

## supplementary crystallographic information

### Crystal data

**Table:**

C_6_H_12_N^+^·Cl^−^	*D*_x_ = 1.276 Mg m^−^^3^
*M**_r_* = 133.62	Mo *K*α radiation, λ = 0.71073 Å
Orthorhombic, *C**m**c*2_1_	Cell parameters from 2988 reflections
*a* = 9.1532 (6) Å	θ = 3.2–30.7°
*b* = 8.7029 (8) Å	µ = 0.45 mm^−^^1^
*c* = 8.7336 (5) Å	*T* = 100 K
*V* = 695.71 (9) Å^3^	Block, colourless
*Z* = 4	0.08 × 0.06 × 0.04 mm
*F*(000) = 288	

### Data collection

**Table:**

Bruker APEX-II CCD diffractometer	777 independent reflections
Radiation source: fine focus sealed tube	769 reflections with *I* > 2σ(*I*)
Graphite monochromator	*R*_int_ = 0.017
φ and ω scans	θ_max_ = 27.0°, θ_min_ = 3.2°
Absorption correction: multi-scan (SADABS; Sheldrick, 2015)	*h* = −11→11
	*k* = −4→11
3239 measured reflections	*l* = −11→10

### Refinement

**Table:**

Refinement on *F*^2^	Hydrogen site location: mixed
Least-squares matrix: full	H atoms treated by a mixture of independent and constrained refinement
*R*[*F*^2^ > 2σ(*F*^2^)] = 0.017	*w* = 1/[σ^2^(*F*_o_^2^) + (0.0282*P*)^2^ + 0.1322*P*] where *P* = (*F*_o_^2^ + 2*F*_c_^2^)/3
*wR*(*F*^2^) = 0.048	(Δ/σ)_max_ < 0.001
*S* = 1.15	Δρ_max_ = 0.21 e Å^−^^3^
777 reflections	Δρ_min_ = −0.12 e Å^−^^3^
47 parameters	Absolute structure: Refined as an inversion twin
1 restraint	Absolute structure parameter: 0.19 (9)

### Special details

**Table:**

Geometry. All esds (except the esd in the dihedral angle between two l.s. planes) are estimated using the full covariance matrix. The cell esds are taken into account individually in the estimation of esds in distances, angles and torsion angles; correlations between esds in cell parameters are only used when they are defined by crystal symmetry. An approximate (isotropic) treatment of cell esds is used for estimating esds involving l.s. planes.
Refinement. Single-crystal data collection was performed using a Bruker Kappa APEX II DUO diffractometer equipped with microfocus optics. Refinement of lattice parameters and subsequent data reduction was carried out with the Bruker *SAINT* software. The crystal structure of 1 was solved and refined using *SHELXT* and *SHELXL*-2014 (Sheldrick, 2015) via the *OLEX2* v.1.2 graphical user interface (Dolomanov *et al.*, 2009). Refined as a 2-component inversion twin.

### Fractional atomic coordinates and isotropic or equivalent isotropic displacement parameters (Å^2^)

**Table:**

	*x*	*y*	*z*	*U*_iso_*/*U*_eq_	
C1	0.62232 (18)	0.2599 (3)	0.5813 (2)	0.0174 (4)	
H1	0.7205	0.2960	0.5547	0.021*	
C2	0.5850 (2)	0.2715 (3)	0.75142 (19)	0.0202 (4)	
H2B	0.6233	0.3655	0.7956	0.024*	
H2A	0.6233	0.1843	0.8078	0.024*	
C6	0.5848 (2)	0.10083 (19)	0.5202 (2)	0.0209 (4)	
H6A	0.6231	0.0210	0.5864	0.025*	
H6B	0.6231	0.0863	0.4176	0.025*	
N7	0.5000	0.3539 (2)	0.5134 (2)	0.0139 (4)	
H7B	0.5000	0.449 (4)	0.548 (3)	0.014 (7)*	
H7A	0.5000	0.347 (4)	0.414 (5)	0.030 (9)*	
Cl1	0.5000	0.31735 (5)	0.15788 (7)	0.01568 (15)	

### Atomic displacement parameters (Å^2^)

**Table:**

	*U*^11^	*U*^22^	*U*^33^	*U*^12^	*U*^13^	*U*^23^
C1	0.0107 (7)	0.0188 (10)	0.0226 (9)	0.0012 (7)	−0.0009 (6)	0.0003 (7)
C2	0.0227 (10)	0.0240 (10)	0.0140 (8)	0.0018 (8)	−0.0063 (7)	0.0029 (7)
C6	0.0239 (9)	0.0157 (9)	0.0232 (9)	0.0046 (6)	−0.0001 (7)	−0.0022 (8)
N7	0.0199 (10)	0.0119 (9)	0.0100 (9)	0.000	0.000	0.0007 (8)
Cl1	0.0213 (2)	0.0142 (2)	0.0116 (2)	0.000	0.000	0.0005 (2)

### Geometric parameters (Å, º)

**Table:**

C1—H1	0.9800	C6—C6^i^	1.553 (4)
C1—C2	1.528 (2)	C6—H6A	0.9700
C1—C6	1.523 (3)	C6—H6B	0.9700
C1—N7	1.508 (2)	N7—C1^i^	1.508 (2)
C2—C2^i^	1.556 (4)	N7—H7B	0.88 (3)
C2—H2B	0.9700	N7—H7A	0.87 (4)
C2—H2A	0.9700		
			
C2—C1—H1	114.5	C1—C6—C6^i^	103.03 (9)
C6—C1—H1	114.5	C1—C6—H6A	111.2
C6—C1—C2	110.50 (19)	C1—C6—H6B	111.2
N7—C1—H1	114.5	C6^i^—C6—H6A	111.2
N7—C1—C2	100.39 (16)	C6^i^—C6—H6B	111.2
N7—C1—C6	100.82 (15)	H6A—C6—H6B	109.1
C1—C2—C2^i^	102.93 (9)	C1—N7—C1^i^	95.91 (18)
C1—C2—H2B	111.2	C1—N7—H7B	111.8 (11)
C1—C2—H2A	111.2	C1^i^—N7—H7B	111.8 (11)
C2^i^—C2—H2B	111.2	C1—N7—H7A	110.6 (14)
C2^i^—C2—H2A	111.2	C1^i^—N7—H7A	110.6 (14)
H2B—C2—H2A	109.1	H7B—N7—H7A	115 (3)
			
C2—C1—C6—C6^i^	70.63 (12)	C6—C1—N7—C1^i^	56.31 (19)
C2—C1—N7—C1^i^	−57.1 (2)	N7—C1—C2—C2^i^	35.24 (15)
C6—C1—C2—C2^i^	−70.56 (14)	N7—C1—C6—C6^i^	−34.89 (12)

Symmetry code: (i) −*x*+1, *y*, *z*.

### Hydrogen-bond geometry (Å, º)

**Table:**

*D*—H···*A*	*D*—H	H···*A*	*D*···*A*	*D*—H···*A*
N7—H7*B*···Cl1^ii^	0.88 (3)	2.25 (3)	3.127 (2)	175 (2)
N7—H7*A*···Cl1	0.87 (4)	2.25 (4)	3.122 (2)	178 (3)

Symmetry code: (ii) −*x*+1, −*y*+1, *z*+1/2.

## References

[bb1] Adams, C., Raithby, P. R. & Davies, J. E. (1997). Private communication (deposition number 100996). CCDC, Cambridge, England.

[bb2] Braun, J. & Schwarz, K. (1930). *Justus Liebigs Ann. Chem.* **481**, 56–68.

[bb3] Britvin, S. N. & Lotnyk, A. (2015). *J. Am. Chem. Soc.* **137**, 5526–5535. 10.1021/jacs.5b01851 25897572

[bb4] Britvin, S. N., Rumyantsev, A. M., Zobnina, A. E. & Padkina, M. V. (2016). *Chem. Eur. J.* pp. 14227–14235. 10.1002/chem.201601637 27531034

[bb5] Britvin, S. N., Rumyantsev, A. M., Zobnina, A. E. & Padkina, M. V. (2017). *J. Mol. Struct.* **1130**, 395–399.

[bb6] Bruker (2015). *APEX2* and *SAINT*. Bruker AXS Inc., Madison, Wisconsin, USA.

[bb7] Carroll, I. F. (2004). *Bioorg. Med. Chem. Lett.* **14**, 1889–1896. 10.1016/j.bmcl.2004.02.007 15050621

[bb8] Cheng, J., Zhang, C., Stevens, E. D., Izenwasser, S., Wade, D., Chen, S., Paul, D. & Trudell, M. L. (2002). *J. Med. Chem.* **45**, 3041–3047. 10.1021/jm0103561 12086489

[bb9] Daly, J. W., Spande, T. F. & Garraffo, H. M. (2005). *J. Nat. Prod.* **68**, 1556–1575. 10.1021/np0580560 16252926

[bb10] Dolomanov, O. V., Bourhis, L. J., Gildea, R. J., Howard, J. A. K. & Puschmann, H. (2009). *J. Appl. Cryst.* **42**, 339–341.

[bb50] Doms, L., Van Hemelrijk, D., Van de Mieroop, W., Lenstra, A. T. H. & Geise, H. J. (1985). *Acta Cryst.* B**41**, 270–274.

[bb11] Dukat, M. & Glennon, R. A. (2003). *Cell. Mol. Neurobiol.* **23**, 365–378. 10.1023/A:1023692705700 PMC11530199 12825833

[bb12] Fitch, A. N. & Jobic, H. (1993). *J. Chem. Soc. Chem. Commun.* pp. 1516–1517.

[bb13] Fletcher, S. R., Baker, R., Chambers, M. S., Herbert, R. H., Hobbs, S. C., Thomas, S. R., Verrier, H. M., Watt, A. P. & Ball, R. G. (1994). *J. Org. Chem.* **59**, 1771–1778.

[bb14] Fraser, R. R. & Swingle, R. B. (1970). *Can. J. Chem.* **48**, 2065–2074.

[bb15] Fun, H.-K., Asik, S. I. J., Chandrakantha, B., Isloor, A. M. & Shetty, P. (2011). *Acta Cryst.* E**67**, o3115. 10.1107/S1600536811044394 PMC3247500 22220118

[bb16] Garraffo, H. M., Spande, T. F. & Williams, M. (2009). *Heterocycles*, **79**, 207–217.

[bb17] Gerzanich, V., Peng, X., Wang, F., Wells, G., Anand, R., Fletcher, S. & Lindstrom, J. (1995). *Mol. Pharmacol.* **48**, 774–782. 7476906

[bb18] Gribkov, D. V., Hultzsch, K. C. & Hampel, F. (2006). *J. Am. Chem. Soc.* **128**, 3748–3759. 10.1021/ja058287t 16536549

[bb19] Groom, C. R., Bruno, I. J., Lightfoot, M. P. & Ward, S. C. (2016). *Acta Cryst.* B**72**, 171–179. 10.1107/S2052520616003954 PMC4822653 27048719

[bb20] Hamama, W. S., Abd El-Magid, O. M. & Zoorob, H. H. (2006). *Heterocycl. Chem.* **43**, 1397–1420.

[bb21] Hori, T., Otani, Y., Kawahata, M., Yamaguchi, K. & Ohwada, T. (2008). *J. Org. Chem.* **73**, 9102–9108. 10.1021/jo801996b 18947252

[bb22] Longobardi, L. E., Mahdi, T. & Stephan, D. W. (2015). *Dalton Trans.* **44**, 7114–7117. 10.1039/c5dt00921a 25797146

[bb23] Macrae, C. F., Bruno, I. J., Chisholm, J. A., Edgington, P. R., McCabe, P., Pidcock, E., Rodriguez-Monge, L., Taylor, R., van de Streek, J. & Wood, P. A. (2008). *J. Appl. Cryst.* **41**, 466–470.

[bb24] Muller, M., Lerner, H.-W. & Bolte, M. (2007). Private communication (deposition number 661061). CCDC, Cambridge, England.

[bb25] Nancy Ghosh, S., Singh, N., Nanda, G. K., Venugopalan, P., Bharatam, P. V. & Trehan, S. (2003). *Chem. Commun.* pp. 1420–1421. 10.1039/b300478c 12841271

[bb26] Ohwada, T., Achiwa, T., Okamoto, I., Shudo, K. & Yamaguchi, K. (1998). *Tetrahedron Lett.* **39**, 865–868.

[bb27] Otani, Y., Nagae, O., Naruse, Y., Inagaki, S., Ohno, M., Yamaguchi, K., Yamamoto, G., Uchiyama, M. & Ohwada, T. (2003). *J. Am. Chem. Soc.* **125**, 15191–15199. 10.1021/ja036644z 14653754

[bb28] Pollini, G. P., Benetti, S., De Risi, C. & Zanirato, V. (2006). *Chem. Rev.* **106**, 2434–2454. 10.1021/cr050995+ 16771455

[bb29] Sheldrick, G. M. (2015). *Acta Cryst.* C**71**, 3–8.

[bb30] Spande, T. F., Garraffo, H. M., Edwards, M. W., Yeh, H. J. C., Pannell, L. & Daly, J. W. (1992). *J. Am. Chem. Soc.* **114**, 3475–3478.

[bb51] Spek, A. L. (2009). *Acta Cryst.* D**65**, 148–155. 10.1107/S090744490804362X PMC2631630 19171970

[bb31] Sullivan, J. F. & Bannon, A. W. (1996). *CNS Drug Rev.* **2**, 21–39.

[bb32] Wang, J., Ma, C., Wu, Y., Lamb, R. A., Pinto, L. H. & DeGrado, W. F. (2011). *J. Am. Chem. Soc.* **133**, 13844–13847. 10.1021/ja2050666 PMC3166227 21819109

[bb33] Westrip, S. P. (2010). *J. Appl. Cryst.* **43**, 920–925.

[bb34] Yogeeswari, P., Sriram, D., Bal, T. R. & Thirumurugan, R. (2006). *Nat. Prod. Res.* **20**, 497–505. 10.1080/14786410600604583 16644549

